# Author Correction: *De novo* phased assembly of the Vitis riparia grape genome

**DOI:** 10.1038/s41597-019-0268-2

**Published:** 2019-10-30

**Authors:** Nabil Girollet, Bernadette Rubio, Céline Lopez-Roques, Sophie Valière, Nathalie Ollat, Pierre-François Bert

**Affiliations:** 1 EGFV, Bordeaux Sciences Agro – INRA – Université de Bordeaux, ISVV, 210 chemin de Leysotte, 33882 Villenave d’Ornon, France; 2 IFV, Institut Français de la Vigne et du Vin, Domaine de l’Espîguette, 30240 Le Grau du Roi, France; 3 INRA, US 1426, GeT-PlaGe, Genotoul, 31326 Castanet-Tolosan, France

Correction to: *Scientific Data* 10.1038/s41597-019-0133-3, published online 19 July 2019

Following publication it was found that co-authors Céline Lopez-Roques, Sophie Valière and Nathalie Ollat were mistakenly omitted from the authorship of this Data Descriptor. The omitted names of these authors, their affiliations, contributions and associated acknowledgements have now been added to both the HTML and PDF versions of the Data Descriptor.

